# Ultrasound-Guided Peripheral Nerve Block as the Sole Anesthetic for Above-Knee Amputation in a Patient With Severe Cardiopulmonary Comorbidities

**DOI:** 10.7759/cureus.110679

**Published:** 2026-06-11

**Authors:** Dilan Shah, Nakosi Stewart, Bright Kim, Matthew Yoder, Anna Ng-Pellegrino, Sharvil Sharvil Sheth

**Affiliations:** 1 Department of Surgery, Medical College of Georgia, Augusta, USA; 2 Department of Surgery, St. Luke's University Health Network, Bethlehem, USA; 3 Department of Anesthesiology, St. Luke's University Health Network, Bethlehem, USA; 4 Department of Anesthesiology and Perioperative Medicine, St. Luke’s Hospital, Bethlehem, USA; 5 Department of Vascular Surgery, St. Luke's University Health Network, Bethlehem, USA

**Keywords:** amputation of knee, cardiopulmonary diseases, limb ischemia, peripheral arterial disease, peripheral nerve block, regional nerve block, ultrasound guided

## Abstract

Above-knee amputation (AKA) carries substantial perioperative risk in patients with advanced cardiopulmonary disease for whom general and neuraxial anesthesia are poorly tolerated. Data supporting peripheral nerve blocks (PNBs) as the primary intraoperative anesthetic remain limited. We present a 79-year-old male with extensive cardiopulmonary and metabolic comorbidities who underwent left AKA for chronic limb-threatening ischemia. Ultrasound-guided sciatic, femoral, obturator, and lateral femoral cutaneous nerve blocks using ropivacaine were performed as the sole anesthetic with intravenous midazolam, achieving complete anesthesia without hemodynamic compromise. The Visual Analog Scale score was 0 postoperatively. The technique was reproduced successfully for contralateral amputation, reinforcing the role of PNBs as a primary anesthetic strategy in high-risk patients.

## Introduction

Above-knee amputation (AKA) represents the endpoint of progressive peripheral artery disease (PAD) that has advanced to chronic limb-threatening ischemia (CLTI), a condition affecting up to 11% of the PAD population and carrying one-year mortality rates that rival those of many malignancies [1,2]. Despite a period of decline through 2013, the annual incidence of AKA has since risen, with approximately 26,000 performed in the United States in 2021, and perioperative mortality ranges from 8% to 23%, substantially exceeding that of below-knee amputations [3,4]. Five-year mortality in elderly patients with CLTI approaches 85% [5].

The standard anesthetic approach (general or neuraxial) is poorly suited to this population. General anesthesia carries well-documented risks, including hemodynamic instability, myocardial injury, acute kidney injury, pulmonary complications, and perioperative dysrhythmias [6]. Myocardial injury after noncardiac surgery (MINS) occurs in 18-20% of patients undergoing major surgery and carries a 10% risk of 30-day mortality [7,8]. Neuraxial anesthesia may be contraindicated in the setting of severe valvular disease, coagulopathy, or anticoagulation and carries its own risk of hemodynamic compromise through sympathetic blockade [9].

Peripheral nerve blocks (PNBs) are increasingly used as adjuncts in lower extremity surgery, but their role as the primary intraoperative anesthetic for AKA remains underutilized. Complete lower extremity anesthesia for AKA requires blockade of four nerves: the sciatic, femoral, lateral femoral cutaneous, and obturator. These nerves collectively provide sensory and motor innervation to the thigh and lower limb. The sciatic nerve supplies the posterior thigh, lower leg, and foot; the femoral nerve innervates the anterior thigh and contributes to knee extension; the obturator nerve provides motor function to the adductor compartment of the medial thigh; and the lateral femoral cutaneous nerve supplies sensation to the anterolateral thigh [10-12]. Because these territories encompass the surgical field and potential sources of intraoperative nociception or movement, blockade of all four nerves is required to achieve complete anesthesia for AKA without general or neuraxial techniques. While individual reports have demonstrated feasibility, widespread adoption has not occurred [13-16]. We describe the successful application of this four-nerve technique as the sole anesthetic for bilateral staged AKA in a patient with prohibitive cardiopulmonary comorbidities.

## Case presentation

**Figure 1:**
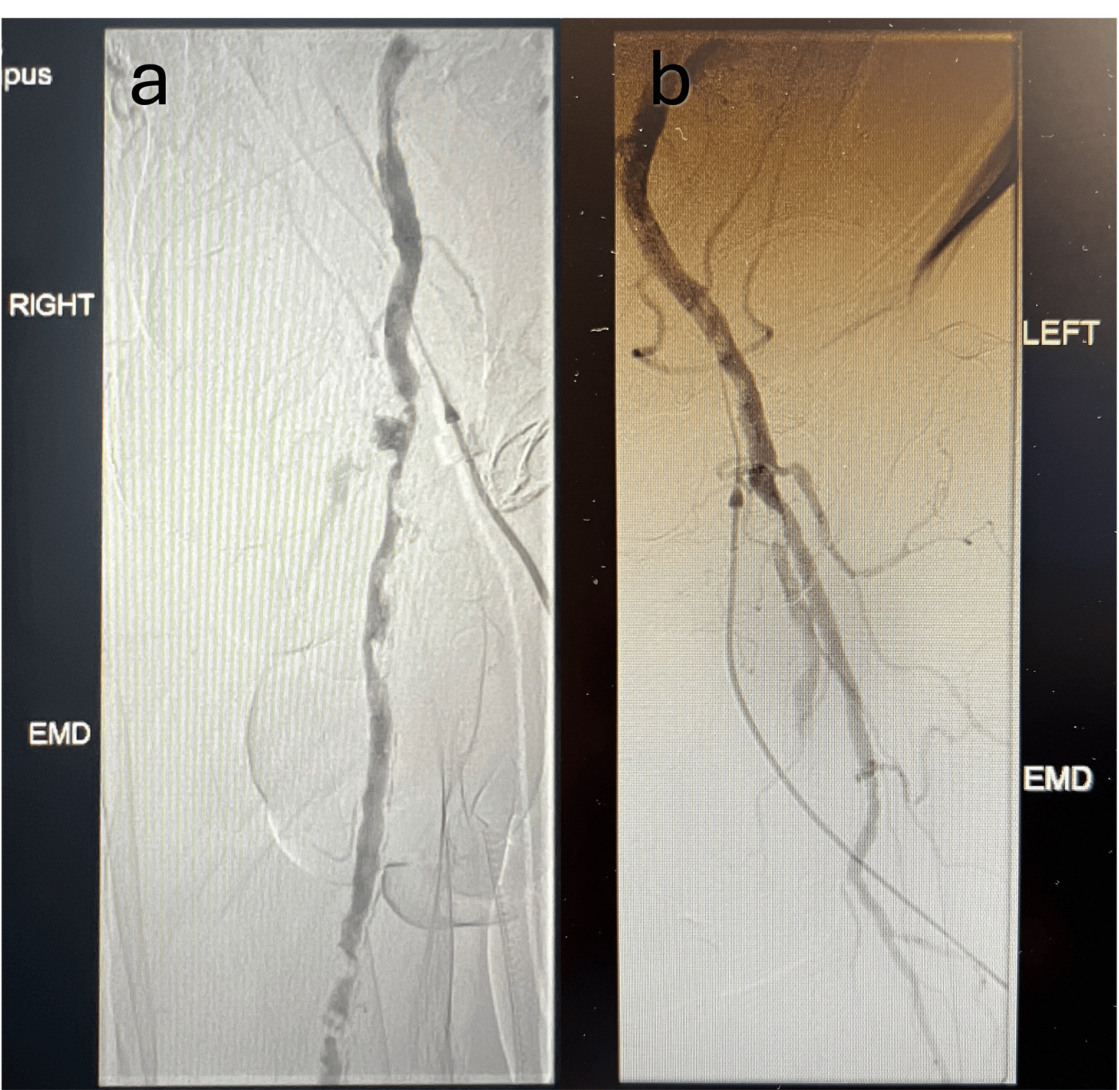
Post-diagnostic angiogram, (a) right lower extremity angiogram and (b) left lower extremity angiogram, both demonstrating diffuse and severe calcific atherosclerotic infrainguinal occlusive disease not amenable to endovascular revascularization.

We present a 79-year-old male with bilateral CLTI and an extensive medical history. Cardiovascular comorbidities included severe peripheral arterial occlusive disease, aortic aneurysmal disease status post EVAR (endovascular aneurysm repair), coronary artery disease status post coronary artery bypass grafting, severe aortic stenosis ineligible for TAVR (transcatheter aortic valve replacement), combined systolic and diastolic heart failure with an ejection fraction of 25%, atrial fibrillation, severe pulmonary hypertension, and refractory hypotension requiring midodrine. Additional comorbidities included COPD (chronic obstructive pulmonary disease) on home oxygen, ESRD (end‑stage renal disease) on hemodialysis following failed renal transplantation, hepatic cirrhosis with refractory ascites requiring weekly paracentesis, idiopathic thrombocytopenic purpura (ITP), and type II diabetes mellitus. Baseline systolic blood pressure was consistently in the 90s mmHg (Table 1). Outpatient evaluation demonstrated dry gangrene of the right forefoot, calf ulceration, and cutaneous changes consistent with critical ischemia. Arterial duplex ultrasonography revealed multisegmental stenosis of the right femoral-popliteal system with tibioperoneal disease and a 1.5 cm popliteal aneurysm; the right ABI (ankle-brachial index) was noncompressible with a toe pressure of 28 mmHg. On the left, mid-SFA (superficial femoral artery) occlusion, tibioperoneal disease, and a 1.2 cm popliteal aneurysm were identified, with a noncompressible ABI and toe pressure of 16 mmHg. Post-diagnostic angiogram demonstrated diffuse and severe calcific atherosclerotic infrainguinal occlusive disease not amenable to endovascular revascularization and was later confirmed by CT angiography (Figure 1).

**Table 1:** Summary of patient profile and comorbidities. ASA (American Society of Anesthesiologists - physical status classification system), HFrEF (heart failure with reduced ejection fraction), EF (ejection fraction), AS (aortic stenosis), TAVR (transcatheter aortic valve replacement), CAD (coronary artery disease), s/p (status post), CABG (coronary artery bypass grafting), HTN (pulmonary hypertension), COPD (chronic obstructive pulmonary disease), ESRD (end-stage renal disease), ITP (immune thrombocytopenic purpura), DM2 (type 2 diabetes mellitus), EVAR (endovascular aneurysm repair).

Patient Profile	
Age/Sex	79-year-old male
Primary Diagnosis	Bilateral chronic limb-threatening ischemia
ASA Classification	ASA 4
Key Comorbidities	
Cardiac	HFrEF (EF 25%), severe AS (TAVR-ineligible), CAD s/p CABG, atrial fibrillation, severe pulmonary HTN, vasopressor-dependent hypotension (midodrine)
Pulmonary	COPD on home oxygen
Renal	ESRD on hemodialysis (failed renal transplant)
Hepatic	Cirrhosis with refractory ascites (weekly paracentesis)
Hematologic	ITP; platelet count 60,000–69,000/μL preoperatively
Metabolic	Type 2 diabetes mellitus
Vascular	Aortic aneurysmal disease s/p EVAR; bilateral popliteal aneurysms

**Figure 2:**
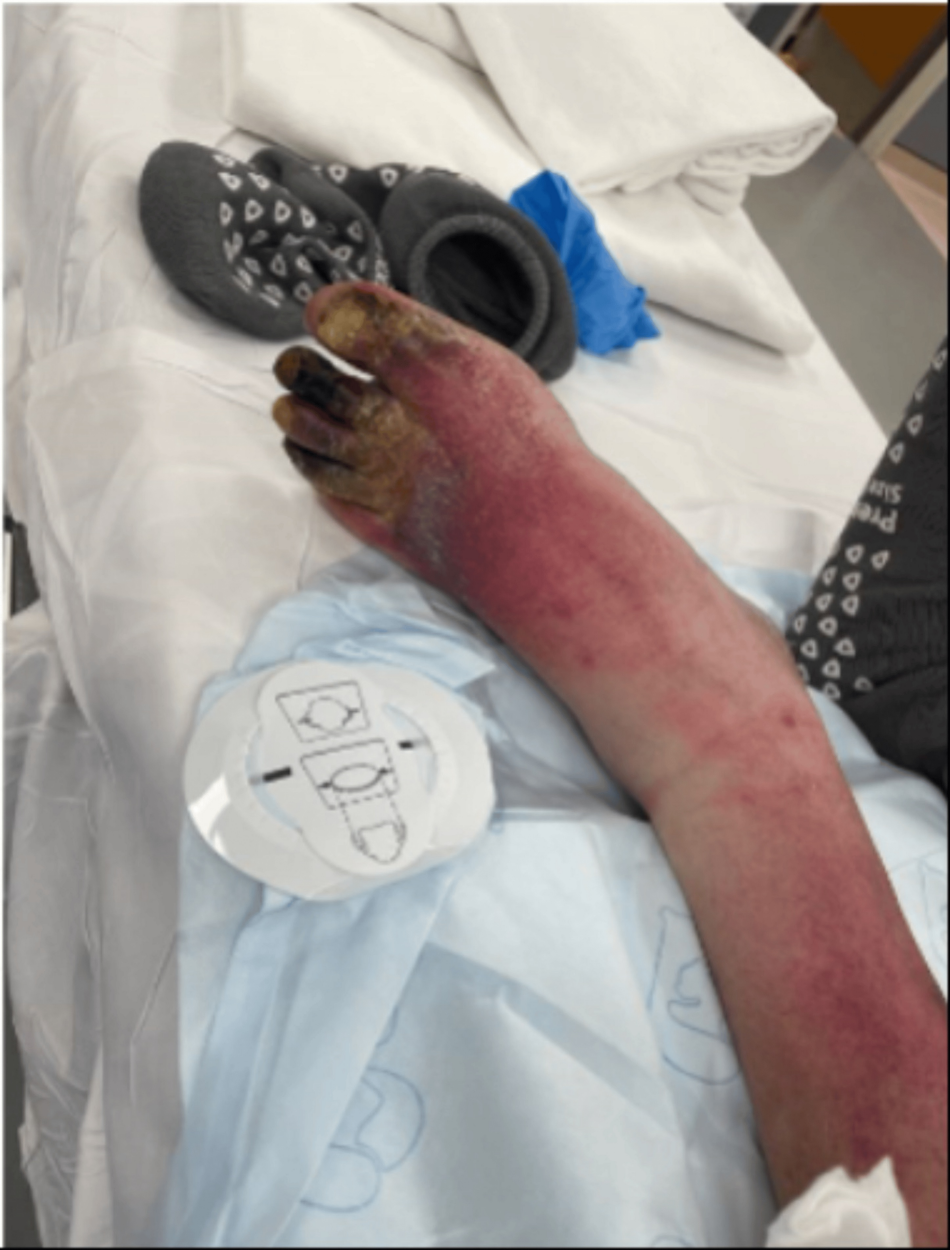
Left lower extremity, anterior view, showing dry gangrenous changes of the second, third, and fourth toes.

**Figure 3:**
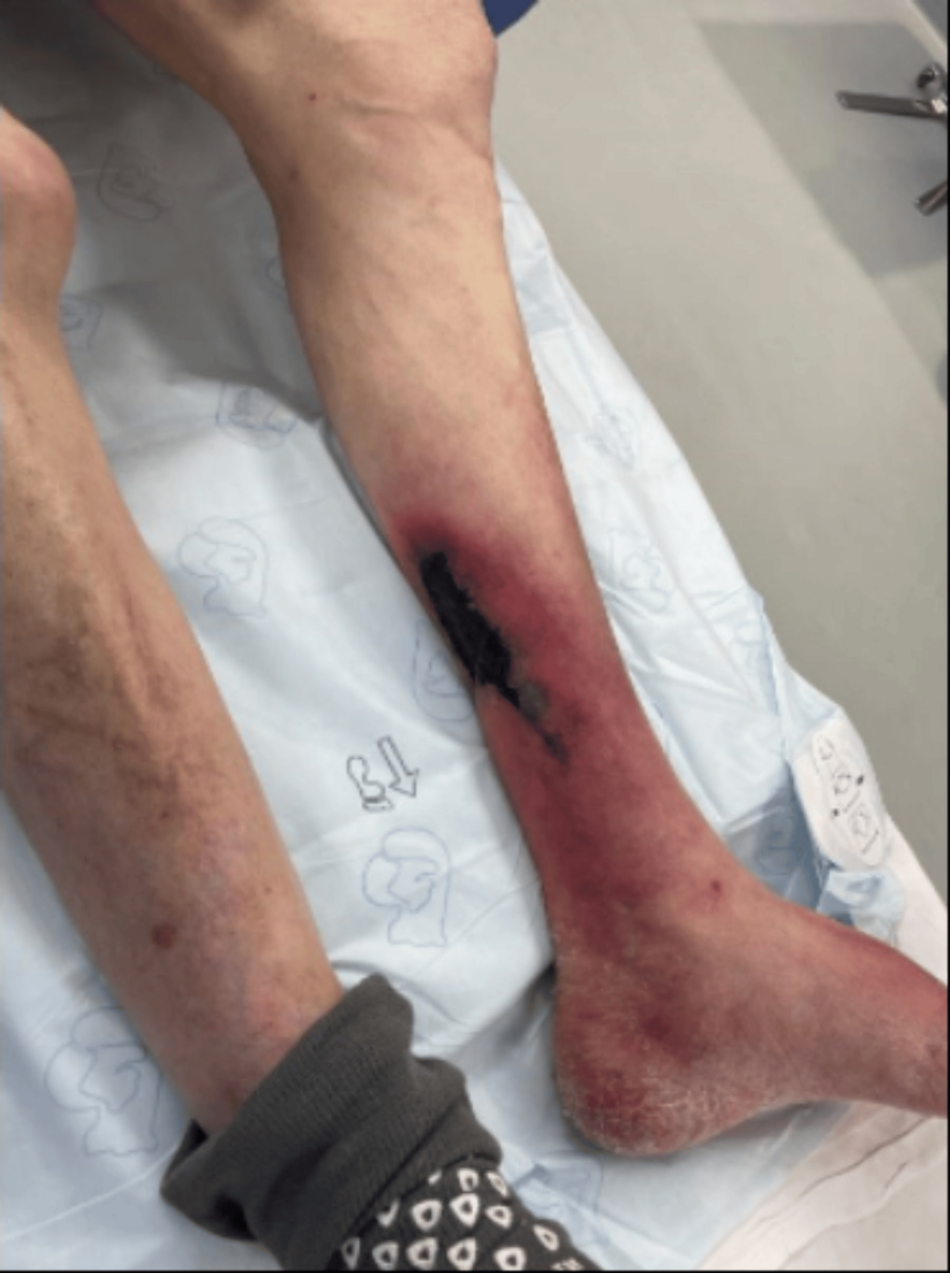
Left lower extremity, lateral view. Eschar with ulceration noted on the posteromedial aspect of the lower leg, measuring approximately 25.1 cm superoinferiorly.

**Figure 4:**
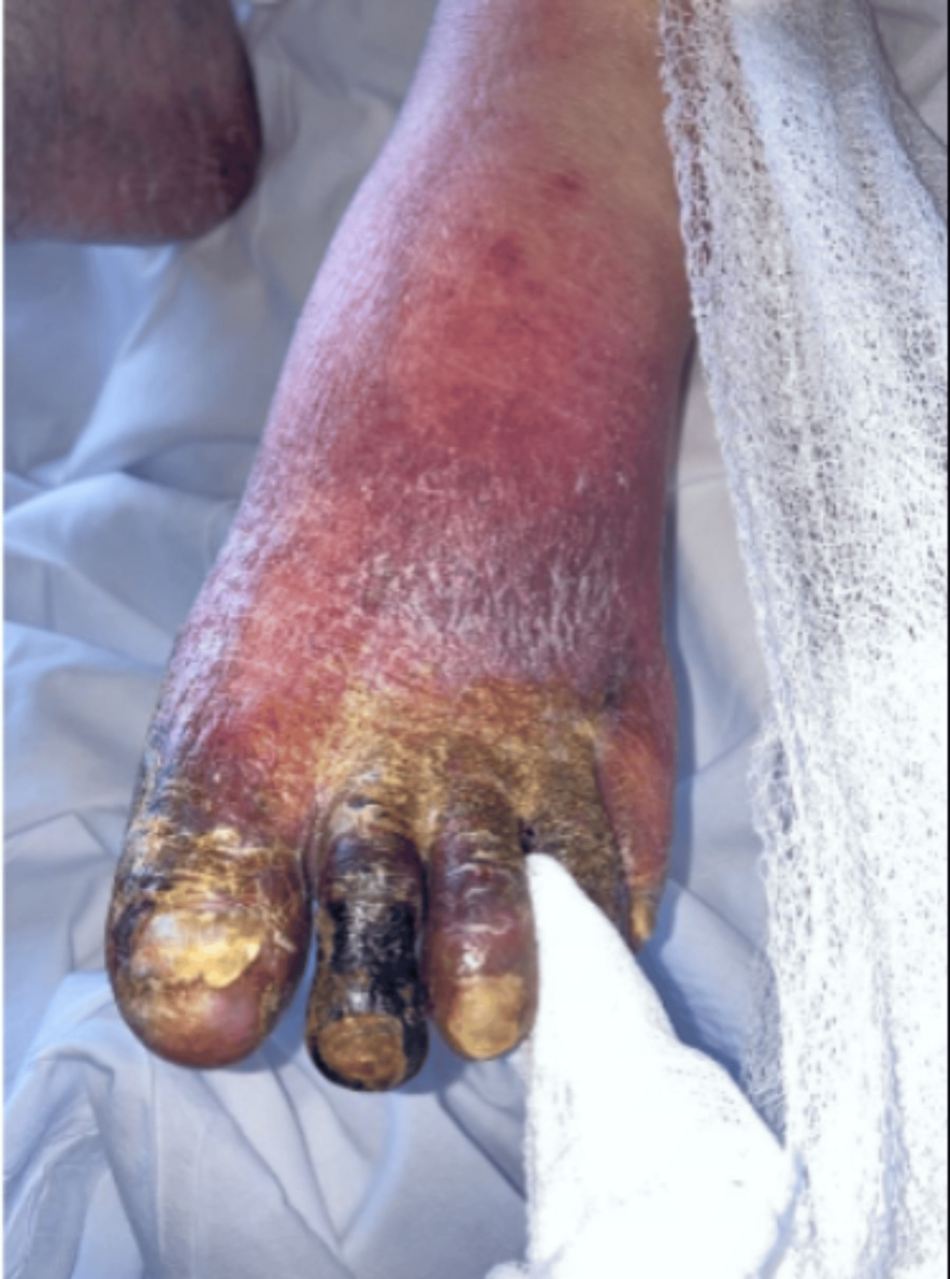
Left foot, viewed anteriorly, showing severe chronic ischemic changes with dry gangrene.

He was admitted electively for definitive management. Examination revealed the left second, third, and fourth toes with dry gangrene and a left calf eschar measuring 25.1 cm (Figures 2-4). Laboratory studies showed BUN 33 mg/dL, creatinine 2.92 mg/dL, and a preoperative platelet count of 69,000/μL. Repeat angiography confirmed the disease was not amenable to endovascular repair. The patient was scheduled for a left AKA after declining palliative care services.

**Figure 5:**
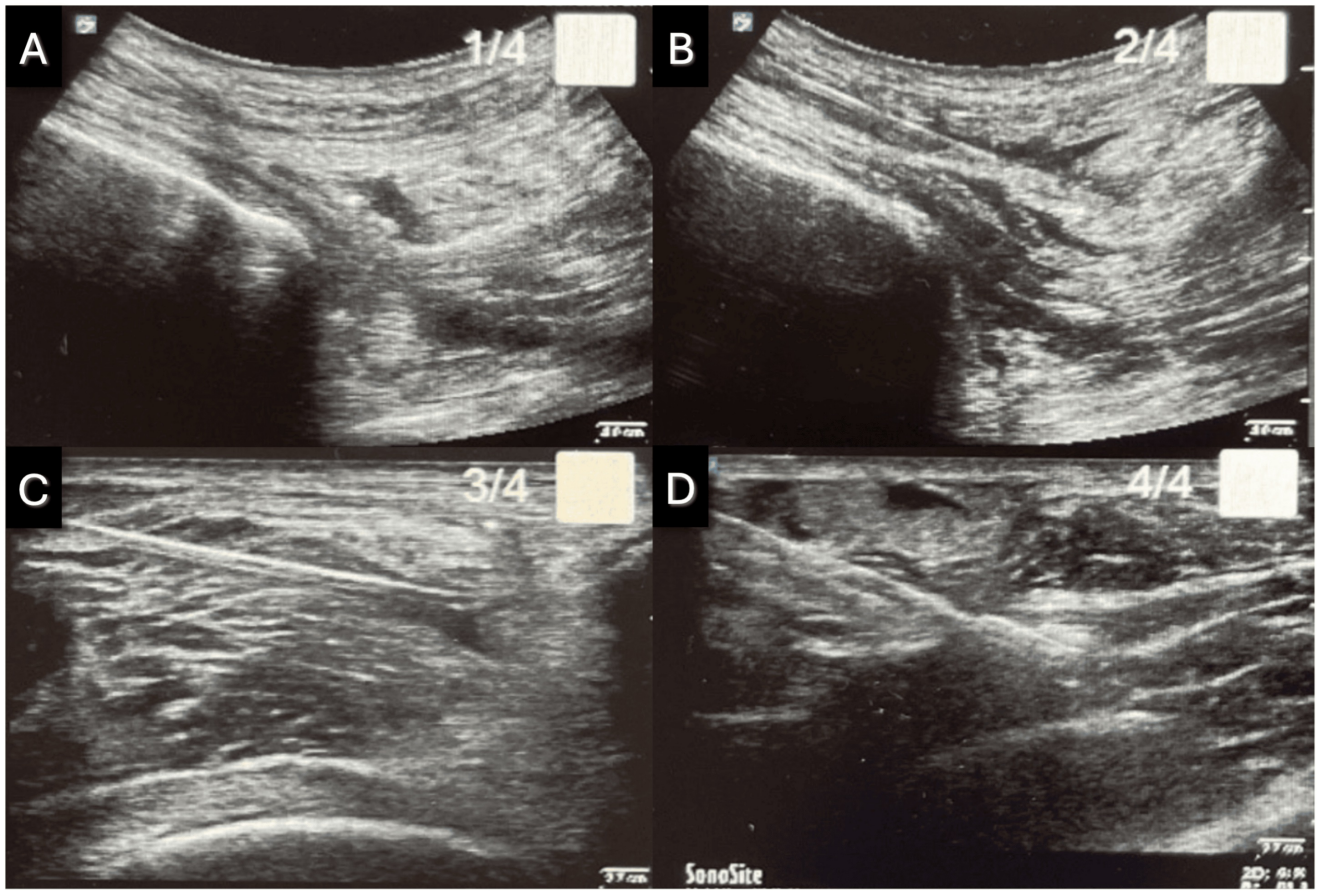
Ultrasound-guided nerve block: (A) left transgluteal sciatic nerve block, (B) left lateral femoral cutaneous nerve block, (C) left femoral nerve block, (D) left obturator nerve block.

Because the patient was classified as ASA (American Society of Anesthesiologists) Physical Status 4 with high cardiovascular risk, the anesthesia team determined peripheral nerve blockade to be the only safe anesthetic modality given the prohibitive risk from both general and neuraxial anesthesia. Ultrasound-guided infragluteal sciatic (20 mL), femoral (12 mL), lateral femoral cutaneous (7 mL), and obturator nerve blocks were performed using 0.5% ropivacaine (Figure 5). Complete sensory and motor block was confirmed in 45 minutes. The patient received 2 mg intravenous midazolam for anxiolysis and tolerated the procedure without respiratory complications. Hemodynamically, systolic blood pressure ranged from 70 to 90 mmHg with a heart rate in the 90s. These values were consistent with the patient’s baseline values; 100 cc of crystalloid was administered throughout the case. His numeric pain rating score was 0 in the PACU. On postoperative day 1, the regional blocks wore off appropriately. The acute pain service was consulted for postoperative pain management and repeated the nerve blocks to help manage phantom limb pain. This produced a satisfactory effect from the patient, further curtailing additional opioid use for the rest of the hospital admission. The patient was discharged to a rehabilitation facility on postoperative day 7.

Three weeks later, the patient was readmitted to manage the right lower extremity dry gangrene. On admission, he was markedly hypotensive secondary to having had 6 L of ascites removed the day prior via paracentesis. Despite presenting with hemodynamic instability, his limb ischemia was deemed critical, prompting the vascular team to proceed with operative management. Once again, the four-nerve block technique was performed on the right limb using 0.5% ropivacaine, with only 1 mg of intravenous midazolam administered for anxiolysis. No intraoperative opioids were administered. The perioperative course was once again uneventful with excellent analgesia. Intraoperative heart rate ranged 100-120 beats per minute, and systolic blood pressure was 60-80 mmHg. A total of 200 cc of crystalloid was administered for the case. Operative time was approximately 40 minutes.

Postoperatively, despite appearing lethargic, the patient remained alert and conversant and was able to answer questions. Nerve blocks wore off appropriately after surgery, and the patient consistently denied pain, only relying on acetaminophen as needed. He remained hypotensive (systolic blood pressure was in the 60-70 mmHg range) despite restarting midodrine and receiving, again, most likely attributed in part to the preceding high-volume paracentesis. Midodrine was restarted postoperatively with 25% albumin. By postoperative day 4, the patient was noted to be more alert, reaching his baseline mentation, but was kept in the ICU due to persistent hypotension.

On postoperative day 5, nephrology determined he was no longer a candidate for dialysis given persistent hypotension. Norepinephrine infusion was started to maintain systolic blood pressure in the 80s. Echocardiography demonstrated severe biventricular failure with a further decline in ejection fraction to 20% and severe right ventricular dysfunction. The patient engaged in goals-of-care discussions with the ICU team, including updating his code status from DNR/DNI to comfort-focused measures with palliative care. Unfortunately, he expired in the early hours of postoperative day 6. Death was most likely attributed to his underlying multiorgan failure and decompensated cardiopulmonary disease; no anesthetic or surgical complications contributed to his clinical deterioration.

Patient consent

Written and verbal informed consent for this case report, including the use of intraoperative imaging, was obtained directly from the patient at the initial procedure. The patient was counseled regarding the nature, purpose, and scope of the report.

## Discussion

This case illustrates that peripheral nerve blockade can serve as the sole anesthetic for an AKA procedure even in patients whose cardiopulmonary function is profoundly compromised. The clinical decision-making here was not a matter of preference but rather the only viable option; an ejection fraction of 25%, severe aortic stenosis ineligible for TAVR, vasopressor-dependent hypotension, severe pulmonary hypertension, thrombocytopenia, and ESRD collectively eliminated both general and neuraxial anesthesia from consideration before the conversation began.

**Figure 6:**
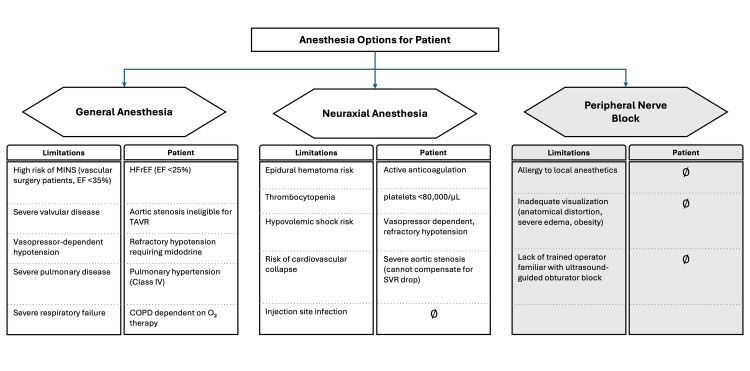
Anesthetic decision framework for above-knee amputation with contraindication mapping of the index patient. MINS (myocardial injury after noncardiac surgery), HFrEF (heart failure with reduced ejection fraction), EF (ejection fraction), TAVR (transcatheter aortic valve replacement), COPD (chronic obstructive pulmonary disease).

General anesthesia was precluded by hemodynamic risk. MINS occurs in approximately 19% of vascular surgery patients and carries a 30-day mortality of 12.5%, with the majority of episodes clinically silent and driven by perioperative oxygen supply-demand mismatch [7]. Neuraxial anesthesia was independently contraindicated: a platelet count of 69,000/μL in the setting of ITP placed neuraxial needle placement beyond accepted safety thresholds, and the sympatholytic effect of spinal anesthesia, which lowers systolic blood pressure in 16-33% of patients, was unacceptable in a vasopressor-dependent patient (Figure 6) [9].

The objective intraoperative data from both procedures support the hemodynamic safety profile of the four-nerve PNB technique in this setting. During the left AKA, intraoperative systolic blood pressure ranged 70-90 mmHg with a heart rate in the 90s, consistent with the patient’s tenuous but stable baseline, and only 100 cc of crystalloid was required. During the right AKA, systolic pressures of 60-80 mmHg and heart rates of 100-120 were observed, attributable in substantial part to intravascular depletion following high-volume paracentesis (6 L) performed the preceding day rather than to an anesthetic effect. Neither procedure required vasopressor escalation attributable to the anesthetic technique, and no opioids were administered intraoperatively or postoperatively during either admission. Total analgesic consumption was limited to as-needed acetaminophen following the left AKA.

One technical point deserves emphasis. The obturator nerve’s sensory distribution lies directly within the field of an AKA. Arising from the ventral rami of L2-L4, it passes through the obturator foramen into the medial thigh and divides into anterior and posterior branches that provide motor innervation to the adductor compartment and sensory innervation to the medial thigh [11,12]. Despite this, the obturator nerve is frequently omitted from peripheral block protocols, likely because its deep course makes it less accessible than the femoral or sciatic nerves and because its contribution to the operative field is underappreciated. In one series, one-third of patients received only femoral and sciatic blocks, with operators citing unfamiliarity with obturator sonoanatomy and concerns about local anesthetic dose constraints as contributing factors [13]. This omission carries meaningful clinical consequences: unblocked adductor muscle activity can produce involuntary thigh movement during surgery, and inadequate medial thigh analgesia necessitates intraoperative supplementation with systemic agents, precisely the agents one aims to avoid in hemodynamically fragile patients [13]. Chandran et al. demonstrated this consequence directly, showing that patients who received the complete four-nerve technique required significantly less intraoperative supplementation than those in whom the obturator was not blocked [13]. Ropivacaine was selected for its established cardiac safety profile; as a pure S-enantiomer, it carries substantially lower cardiotoxic potential than racemic bupivacaine [17,18].

The broader literature supports PNBs as a viable primary anesthetic for AKA. A retrospective cohort of 1,916 ACS-NSQIP (American College of Surgeons National Surgical Quality Improvement Program) patients found no mortality difference between general and regional anesthesia, though peripheral block was not isolated from neuraxial techniques [4]. A 2023 systematic review reached similar conclusions [15]. Bech et al. described four patients with severe cardiac insufficiency who maintained hemodynamic stability under PNBs alone [16], and Karm et al. and Shamim et al. extended this approach to anticoagulated patients for whom neuraxial anesthesia was explicitly contraindicated [14,19]. The present case adds to this evidence base by documenting successful bilateral staged AKA in a patient whose cumulative comorbidity profile exceeds that reported in any prior series, with reproducibility confirmed across two separate procedures.

Despite a growing body of favorable evidence, PNBs as the primary anesthetic for AKA remain underutilized in clinical practice. The reasons are multifactorial and include practice inertia favoring general anesthesia, training imbalances in regional anesthesia techniques, particularly in nonacademic environments, unfamiliarity with ultrasound-guided obturator block, and the absence of prospective randomized data or formal guidelines specifically endorsing the four-nerve technique as a standalone approach [4,16]. Recognizing and addressing these barriers is a necessary step before the technique can achieve broader adoption.

Limitations

The principal limitations of this report are its single-case design and the clinical rather than dermatomal assessment of block completeness. Postoperative analgesia required repeat block performance on day 1 as single-injection coverage resolved; prospective consideration of perineural catheter placement for continuous local anesthetic infusion may provide more durable analgesia in future cases of comparable complexity.

## Conclusions

This case demonstrates that PNBs can serve as a safe and effective primary anesthetic for AKA in patients for whom general and neuraxial anesthesia are contraindicated. Successful bilateral replication in a patient with significant cardiopulmonary comorbidities and the absence of anesthetic-attributable complications helps reinforce the reliability and durability of the PNB approach. These findings add to the existing literature, suggesting that PNB warrants early consideration in preoperative planning rather than being a last resort in these high-risk patients. Additional studies involving prospective, multicenter data can be used to further support the study and expand generalizability across a wide variety of clinical settings. As institutional familiarity with the four-nerve technique grows and prospective data accumulate, PNBs may offer a meaningful reduction in perioperative morbidity for a population in whom current anesthetic options are severely constrained.
